# GABARAPL1 suppresses metastasis by counteracting PI3K/Akt pathway in prostate cancer

**DOI:** 10.18632/oncotarget.13879

**Published:** 2016-12-10

**Authors:** Wei Su, Shibao Li, Xiaofan Chen, Lingyu Yin, Ping Ma, Yingyu Ma, Bing Su

**Affiliations:** ^1^ Department of Orthopedics, The Third Affiliated Hospital, Xinxiang Medical University, Xinxiang, Henan, China; ^2^ Department of Laboratory Medicine, The Affiliated Hospital of Xuzhou Medical College, Xuzhou, Jiangsu, China; ^3^ Biomedical Research Institute, Shenzhen-PKU-HKUST Medical Center, Shenzhen, Guangdong, China; ^4^ Department of Pharmacology and Therapeutics, Roswell Park Cancer Institute, Buffalo, NY, USA; ^5^ Xinxiang Key Lab of Translational Cancer Research, The Third Affiliated Hospital, Xinxiang Medical University, Xinxiang, Henan, China; ^6^ Department of Epidemiology and Environmental Health, School of Public Health and Health Professions, University at Buffalo, Buffalo, NY, USA

**Keywords:** GABARAPL1, prostate cancer, metastasis, Akt, FOXOs

## Abstract

Metastasis remains the primary cause of prostate cancer (CaP)-related death. Using a genome wide shRNA screen, we identified GABARAPL1 as a potential CaP metastasis suppressor. GABARAPL1 mRNA levels inversely correlate with the invasive potential of a panel of human CaP cell lines. Lower mRNA levels correlate with higher Gleason scores in clinical CaP tumor samples. Moreover, Kaplan-Meier curves analysis showed that GABARAPL1 down-regulation in cancer tissues is associated with decreased disease-free survival in CaP patients. Knockdown of GABARAPL1 in human LNCaP cells results in increased invasion *in vitro* and lymph node metastasis *in vivo*. Vice versa, ectopic expression of GABARAPL1 decreases the invasiveness of CWR22Rv1 cells. Our previous *in vitro* shRNA screening identified FOXO4, a PI3K/Akt-inactivating downstream target, as a potential CaP metastasis suppressor. We show here that silencing FOXOs leads to reduced GABARAPL1 expression and enhanced invasion in LNCaP cells. Transfection of constitutively-activated Akt (myr-Akt) increased the invasion of LNCaP cells, which is associated with the inactivation of FOXOs and decreased GABARAPL1 expression. Indeed, forced expression of GABARAPL1 reversed the increased invasiveness of LNCaP/myr-Akt cells. Finally, immunohistochemistry analysis shows that Akt phosphorylation is negatively correlated with GABARAPL1 expression in human CaP tissues. Taken together, our data indicate that the suppression of FOXOs-GABARAPL1 signaling by Akt is an important mechanism for CaP progression and metastasis.

## INTRODUCTION

Metastasis remains the primary cause of prostate cancer (CaP)-related death, which is the second leading cause of cancer deaths in men in the U.S. [1]. Unfortunately, no effective therapy is currently available [2, 3].

PI3K/Akt pathway is a major contributor to CaP progression [4, 5]. In 42% of the primary CaP lesions and 100% of the metastatic tumors, PI3K/Akt pathway exhibits alterations (mutations/deletions, copy number variations, differential gene expression) in one or more components [6]. Because of the critical role of the PI3K/Akt pathway in promoting cancer progression, many inhibitors targeting this pathway are invented and tested in clinical trials [7]. Unfortunately, the therapeutic efficacy of these inhibitors remains modest [8]. Identifying genes that regulate the metastatic cascade will lead to novel and more effective treatment for the deadly metastatic CaP and prolong survival of CaP patients.

In order to understand the genetic components necessary for CaP metastasis, we used a high throughput shRNA screening approach to identify potential metastasis suppressors in the LNCaP orthotopic CaP model *in vivo*. Using DNA sequence analysis and the BLAST databank search, we identified gamma-aminobutyric acid (GABA) A receptor-associated protein-like 1 (GABARAPL1) as a metastasis suppressor candidate. GABARAPL1, also known as autophagy-related 8 (ATG8) or Glandular epithelial cell protein 1 (GEC1), belongs to the GABARAP family [9]. GABARAPL1 plays important roles in protein interaction and transportation, as well as autophagy, cell proliferation and tumor progression [9].

Previously, we used this functional shRNA screening approach in an invasion assay *in vitro* and identified FOXO4, a PI3K/Akt-inactivating downstream target, as a potential CaP metastasis suppressor [10, 11]. Interestingly, several groups showed that GABARAPL1 expression is regulated by FOXOs [12–14], indicating that GABARAPL1 is a downstream target of FOXOs. Moreover, a recent study showed that Akt decreased the expression of GABARAPL1 [15]. Therefore, we hypothesized that the anti-metastatic GABARAPL1 may be involved in PI3K/Akt pathway. We next sought to determine the effect of activated Akt on the expression of FOXOs and GABARAPL1 and cell invasion in LNCaP cells. Our data strongly indicate that the suppression of FOXOs-GABARAPL1 signaling by Akt may be an important mechanism for CaP progression and metastasis.

## RESULTS

### Genome-wide shRNA screening in LNCaP orthotopic CaP model identified GABARAPL1 as a potential metastasis suppressor

**Figure 1:**
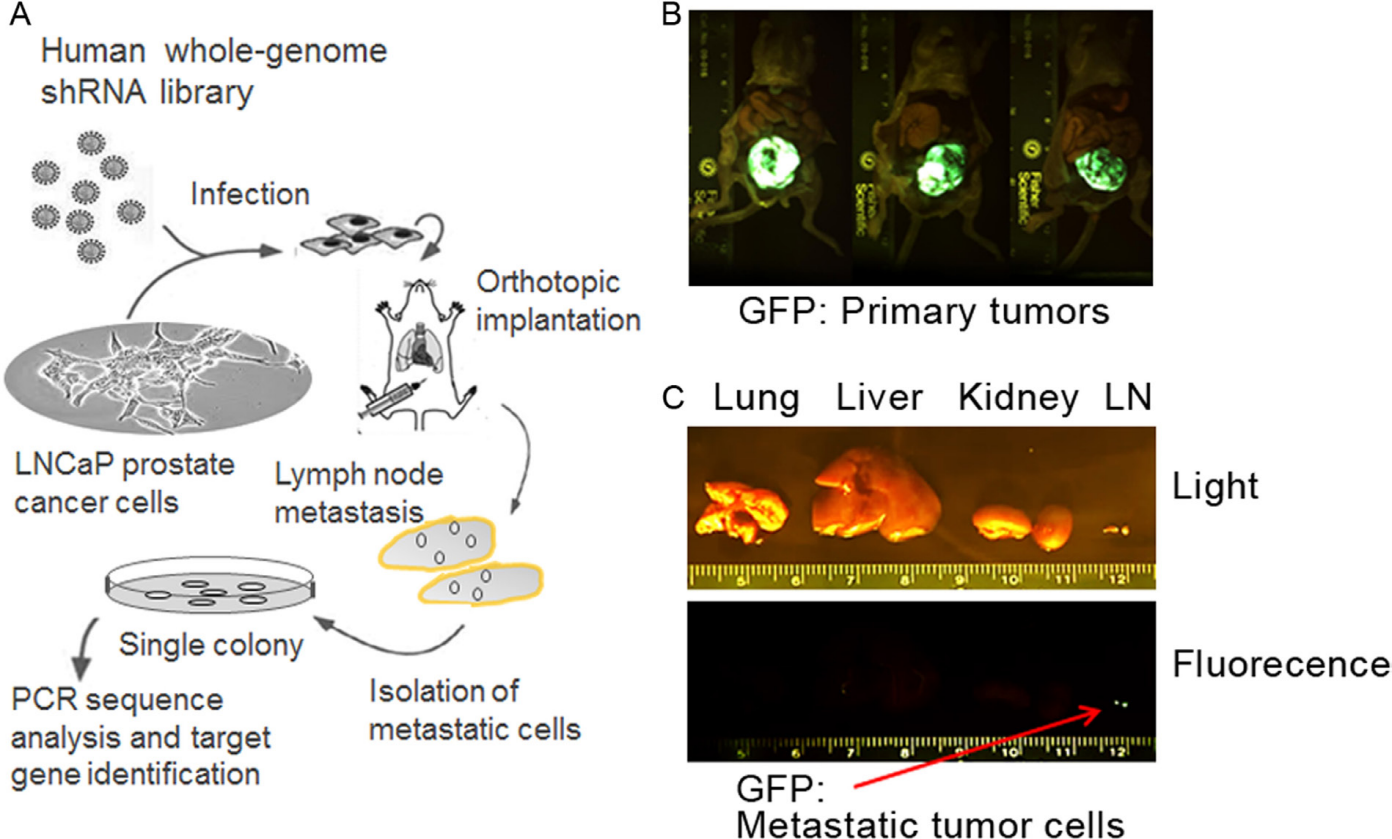
Loss-of-function selection for metastasis-suppressor genes in the LNCaP CaP orthotopic xenograft model (**A**) A schematic summary of the high-throughput genome-wide shRNA screening approach to identify genes suppressing regional lymph node metastasis in the LNCaP orthotopic metastatic model. (**B**) Sustained GFP fluorescence in the primary tumors formed after the injection of LNCaP cells infected with the DECODE (OpenBiosystems) pooled pGIPZ lentivirus library encoding human shRNAs. (**C**) A GFP fluorescence image of lymph node metastasis from one representative mouse. Red arrow indicates metastasis in the GFP image.

To better understand the genetic components necessary for CaP metastasis, we used a high throughput genome-wide shRNA screening approach to identify genes suppressing lymph node metastasis in the LNCaP orthotopic CaP model. A schematic summary of the *in vivo* shRNA library screening of potential metastasis suppressor genes is presented in Figure 1A. We aimed to select for metastatic cells in regional lymph nodes from low invasive LNCaP cells, which were infected with GFP-labeled lentivirus encoding human genome-wide shRNA library (~10,000 shRNAs). Stable clones were established and orthotopically injected to the prostates of male nude mice that have embedded testosterone pellets in the flanks. The growth of the primary tumors and pelvic local lymph node metastasis was assessed 10 weeks later by a bioluminescence imager. The primary tumors and lymph node metastasis were visible (Figure 1B–1C). Metastasis was not observed in lungs, livers and kidneys (Figure 1C). Cells were isolated from the lymph nodes and puromycin resistant metastatic LNCaP cells were selected *in vitro*. Single colonies were established and PCR sequencing analysis with the bar codes and shRNA sequences followed by the BLAST databank searching (http://blast.ncbi.nlm.nih.gov/Blast.cgi) was performed to identify the genes that were silenced in these metastatic LNCaP cells by the shRNAs. These genes include GABA(A) receptor-assocated protein like 1 (GABARAPL1), Leucine proline-enriched proteoglycan (leprecan)1(LEPRE1), Myosin IIIB (MYO3B), zinc finger protein 763 (ZNF763), tRNA aspartic acid methyltransferase 1 (TRDMT1), Opoid growth factor receptor-like 1 (OGFRL1), and Dyslexia susceptibility 1 candidate 1 (DYX1C1) (Table 1). Given the growing understanding of the association between autophagy and cancer progression and metastasis [16, 17], we selected GABARAPL1 to further investigate its potential as a metastasis suppressor.

**Table 1:** Candidate metastasis suppressor genes identified from the genome-wide shRNA screening

Gene symbol	Name	Accession	Biological functions
LEPRE1	Leucine proline-enriched proteoglycan(leprecan)1	NM_001042411, NM_019782, NM_019783, NM_022356	cell growth, protein metabolic process, negative regulation of cell proliferation, protein metabolic process
MYO3B	Myosin IIIB	NM_001083615, NM_138995	nucleotide binding, motor activity, actin binding, protein serine/threonine kinase activity, ATP binding, transferase activity
ZNF763	zinc finger protein 763	AK092240, NM_001012753, XM_001131183	nucleic acid binding, DNA binding, zinc ion binding, metal ion binding
TRDMT1	tRNA aspartic acid methyltransferase 1	NM_004412, NM_176081, NM_176083	DNA binding, RNA binding, DNA (cytosine-5-)-methyltransferase activity, methyltransferase activity, tRNA (cytosine-5-)-methyltransferase activity, transferase activity
GABARAPL1	GABA(A) receptor-assocated protein like 1	NM_031412	protein binding, beta-tubulin binding, GABA receptor binding
OGFRL1	Opoid growth factor receptor-like 1	NM_024576	receptor activity
DYX1C1	Dyslexia susceptibility 1 candidate 1	NM_001033559, NM_130810	binding

### GABARAPL1 is downregulated in human metastatic CaP cell lines and CaP tumor tissues

**Figure 2:**
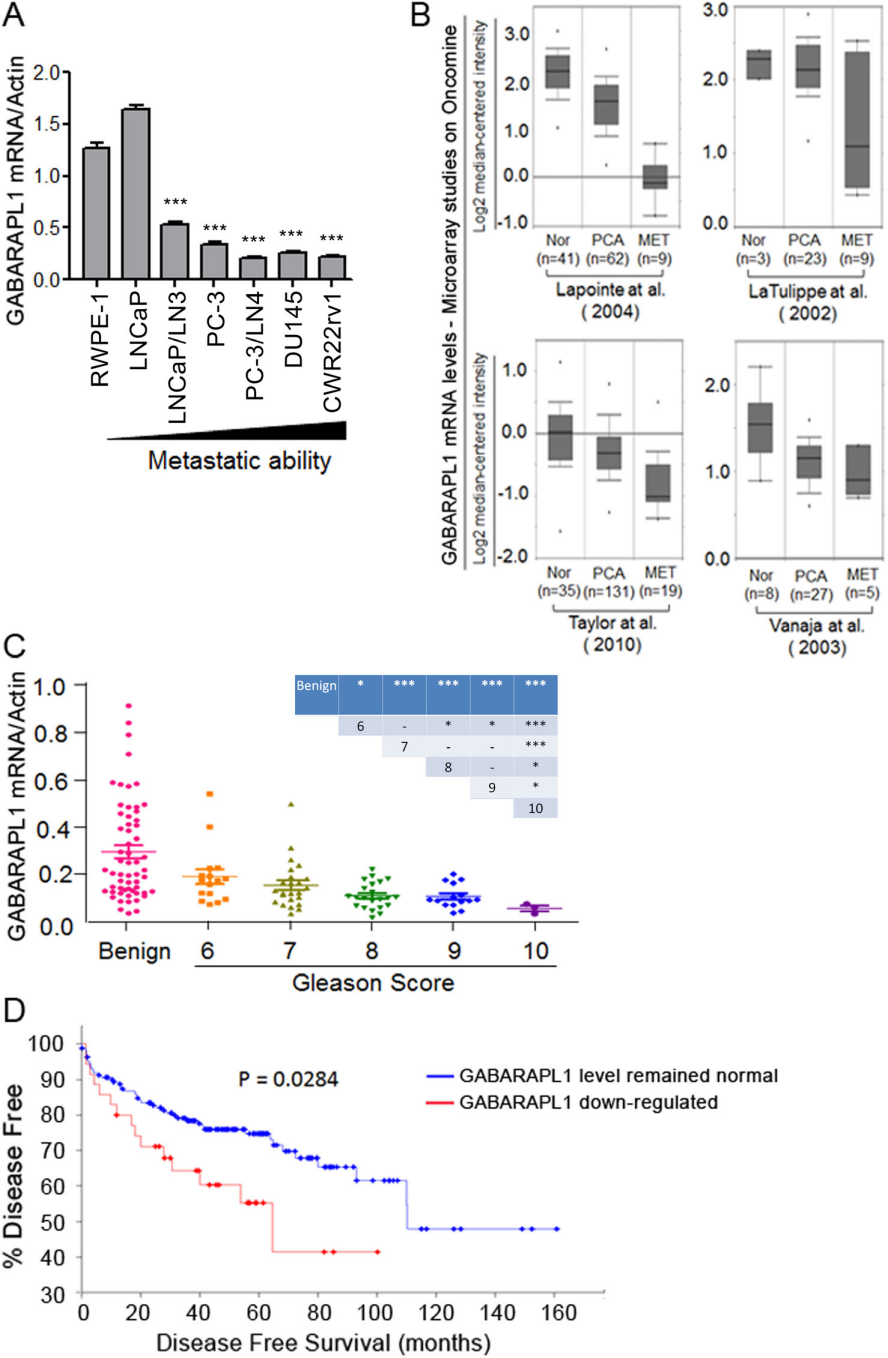
GABARAPL1 expression is inversely associated with invasiveness and metastatic in CaP cells and tissues (**A**) GABARAPL1 expression in immortalized prostate epithelial cells RWPE-1 and CaP cell lines with different metastatic ability is assessed by qRT-PCR. Error bars, SE of three independent qRT-PCR assays. ***P < 0.001; cells indicated vs. RWPE-1 cells. (**B**) GABARAPL1 mRNA expression levels in normal (Nor), primary cancer (PCA) or metastases (MET). CaP tissues obtained from four studies available on the Oncomine website: Lapointe et al. [39], LaTulippe et al. [40], Taylor et al. [6], and Vanaja et al. [41]. (**C**) The expression of GABARAPL1 mRNA in a cohort of 80 CaP tumor tissue samples, categorized by their Gleason scores, and 59 benign prostate tissue samples was assessed by qRT-PCR. *P* values between specific two groups were showed in the inserted table: *P < 0.05; **P < 0.01; ***P < 0.001. (**D**) Kaplan-Meier plot analysis (http://www.cbioportal.org/public-portal/) of disease free vs. time in 174 CaP cases from a study conducted by Taylor et al. [6] is presented.

The expression levels of metastasis suppressor genes are often lower in highly metastatic tumor cells compared to poorly metastatic or non-metastatic tumor cells or normal cells [18]. To examine whether the expression pattern of GABARAPL1 is reversely related to metastasis potential, we first assessed GABARAPL1 mRNA expression in six CaP cell lines with various metastatic tendency and an immortalized human primary prostate epithelial cell RWPE-1 by qRT-PCR. GABARAPL1 expression is high in RWPE-1 and LNCaP cells (Figure 2A). More importantly, GABARAPL1 expression is inversely correlated with the metastatic ability of CaP cells (Figure 2A). LNCaP/LN3 and PC-3/LN4, lymph node metastatic variants, showed markedly lower expression of GABARAPL1 compared to their parental lines LNCaP and PC-3, respectively (Figure 2A). Highly metastatic human CaP cell lines DU145 and CWR22Rv1 both have very low levels of GABARAPL1 (Figure 2A). The differences between RWPE-1 cells and CaP cell lines except for LNCaP cells are statistically significant (*P* < 0.001). Further, data mining in the Oncomine database (http://www.oncomine.org) including 4 individual studies revealed that GABARAPL1 is significantly down-regulated in metastatic CaP compared to primary tumor samples, and normal prostate tissue samples have the highest GABARAPL1 expression (Figure 2B).

To further investigate the observed inverse relationship of GABARAPL1 expression and metastasis in CaP cell lines, GABARAPL1 mRNA expression was examined in 80 CaP tumor tissues and 59 benign prostate tissue samples from benign prostatic hyperplasia (BPH) patients by qRT-PCR. Overall, GABARAPL1 mRNA expression levels in CaP tumor tissues were significantly lower than those in the benign prostate tissues (*P* < 0.001) (Figure 2C). Among the CaP tumor tissues, a progressive loss of GABARAPL1 was found with increased Gleason scores (Figure 2C). Moreover, analysis of data from a published study including 174 CaP cases [6], available on the *cbioportal* website (http://www.cbioportal.org/public-portal/), revealed that 32 cases (18%) that displayed GABARAPL1 downregulation in CaP tumor tissues had shorter disease free time compared with the 142 cases (82%) whose GABARAPL1 expression levels were not altered in CaP tumor tissues (Figure 2D). These data strongly indicate that the loss of GABARAPL1 correlates with the aggressiveness of CaP.

### Knock-down of GABARAPL1 promotes LNCaP cell invasion

**Figure 3:**
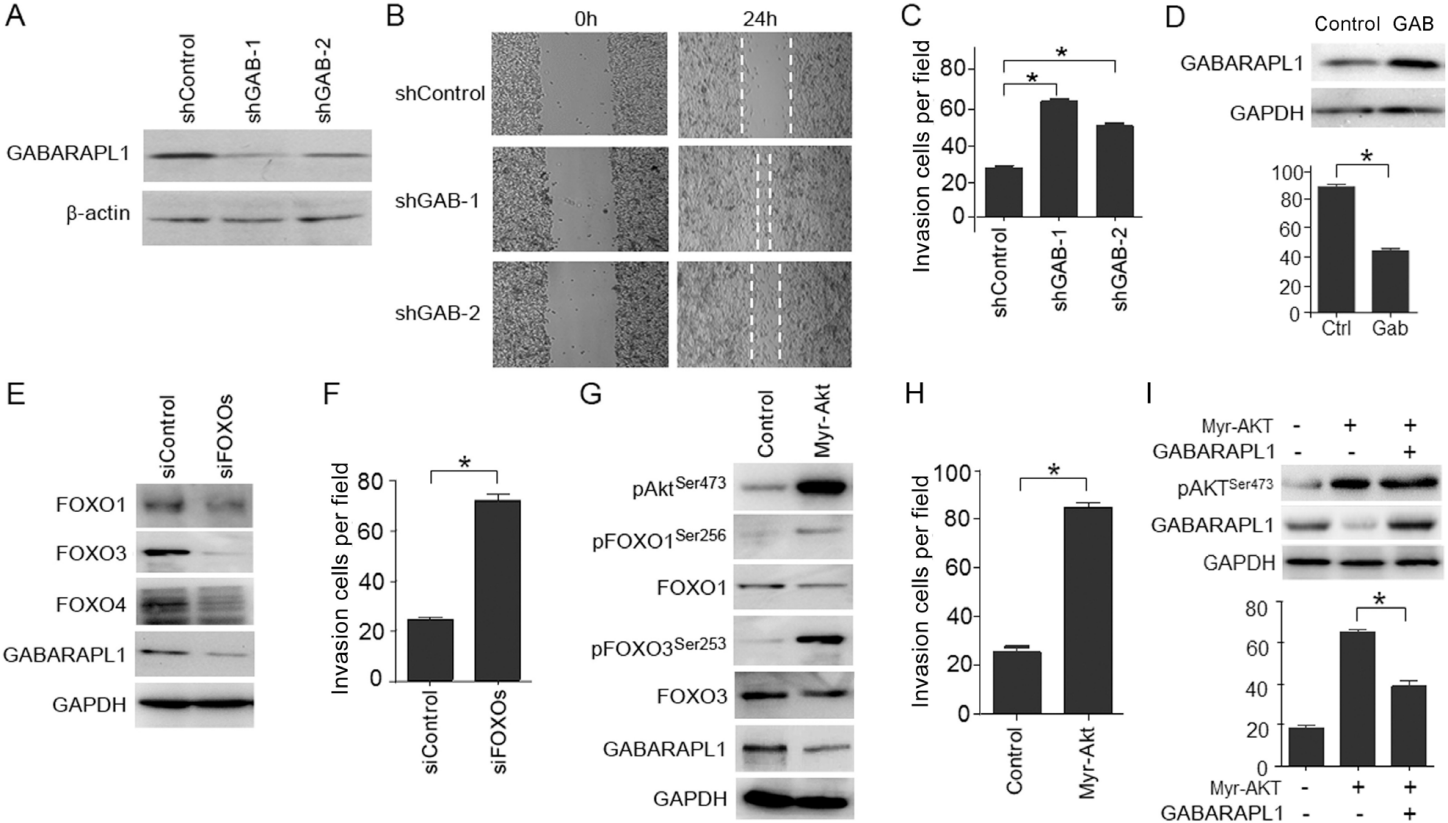
LNCaP cell invasion is promoted by Akt-mediated inactivation of FOXOs and the expression of FOXOs-induced gene GABARAPL1 (**A**) GABARAPL1 expression was knocked down by shRNAs and validated using IB analysis. β-actin was the internal control. (**B**) Wounds were introduced by scratching confluent monolayers of LNCaP cells transfected with shControl, shGAB-1 or shGAB-2. Migration was monitored by light microscopy at 0 and 24 h. (**C**) Matrigel-based invasion assay was performed in LNCaP cells transfected with shControl, shGAB-1 or shGAB-2. The cell numbers per field were counted and the results summarized in a bar graph. Error bars, S.E. of triplicate experiments. *P < 0.05. (**D**) Ectopic expression of wild type-GABARAPL1 decreases CWR22Rv1 invasiveness. Protein expression of GABARAPL1 was assessed by IB analysis (upper panel). The effect of overexpression of GABARAPL1 on Matrigel invasiveness was quantified (lower panel). Error bars, S.E. of triplicate experiments. *P < 0.05. (**E**) FOXOs (FOXO1, FOXO3 and FOXO4) were knocked down by siRNA transfections in LNCaP cells. Protein expression of FOXOs and GABARAPL1 was assessed by IB analysis. GADPH was the internal loading control. (**F**) LNCaP cells with FOXOs knockdown were subjected to Matrigel-based invasion assays. The cell numbers per field were counted and the results summarized in a bar graph. Error bars, S.E. of triplicate experiments. *P < 0.05. (**G**) LNCaP cells were transiently transfected with vectors harboring constitutively activated Akt (myr-Akt). Expression of phosphor-Akt (Ser473), phosphor-FOXO1 (Ser256), FOXO1, phosphor-FOXO3 (Ser253), FOXO3, and GABARAPL1 was assessed by IB analysis. GADPH was the loading control. (**H**) LNCaP cells transfected with myr-Akt or control vector were subjected to Matrigel-based invasion assays. The cell numbers per field were counted and the results summarized in a bar graph. Error bars, S.E. of triplicate experiments. *P < 0.05. (**I**) LNCaP cells were transiently transfected with control vector, myr-Akt or myr-Akt plus GABARAPL1. Expression levels of pAkt (Ser473) and GABARAPL1 were assessed by IB analysis. GAPDH was the loading control (upper panel). Matrigel invasion assay of LNCaP cells expressing control, myr-Akt or myr-Akt plus GABARAPL1 was presented in the lower panel. Error bars, S.E. of triplicate experiments. *P < 0.05.

To determine whether the down-regulation of GABARAPL1 contributes to the highly metastatic capacity of CaP cells, LNCaP cells were transiently infected with two different shRNAs against GABARAPL1 and used to evaluate the impact on cell motility and invasiveness. Successful knockdown of GABARAPL1 protein expression was validated by Western blot analysis (Figure 3A). We next examined whether the knockdown of GABARAPL1 affects LNCaP cell motility using the scratch “wound” healing assay. LNCaP cells have poor motility and thus the wounded gap was only partially covered by shControl-transfected LNCaP cells in 24 h (Figure 3B). In contrast, either of the two shGABARAPL1-transfected LNCaP cells covered almost the entire damaged area by 24 h (Figure 3B), indicating that knockdown of GABARAPL1 promotes CaP cell motility. To further examine the involvement of GABARAPL1 in the invasion of CaP cells, LNCaP cells infected with shRNA-GABARAPL1 were used in the Matrigel-based invasion assay. shRNA-GABARAPL1 infection resulted in significantly (*P* < 0.05) increased invasion in LNCaP cells compared to control shRNA infection (Figure 3C). Conversely, overexpression of wild type-GABARAPL1 resulted in decreased invasiveness in CWR22Rv1 cells (Figure 3D). Taken together, these observations indicate that GABARAPL1 may be a potential metastasis suppressor.

### Loss of FOXOs down-regulates GABARAPL1 and promotes invasion in LNCaP cells

To determine whether there is a relationship between FOXOs, GABARAPL1 and CaP invasion, LNCaP cells were transiently transfected with siRNA against three FOXOs (FOXO1, FOXO3 and FOXO4) or scrambled control siRNA, and subjected to Matrigel-based invasion assays. Three FOXO proteins were successfully knocked down as validated by Western blot analysis (Figure 3E). The knockdown of FOXOs resulted in decreased expression of GABARAPL1 (Figure 3E) and significant increase in the invasiveness of LNCaP cells (*P* < 0.05) (Figure 3F). These data suggest that FOXOs may suppress the invasiveness of LNCaP cells through regulating GABARAPL1 expression.

### Activation of Akt inhibits FOXOs and their target gene GABARAPL1 and promotes CaP invasion

PI3K/Akt pathway is a major contributor to CaP progression [4, 5]. When PI3K/Akt pathway is activated in CaP cells, Akt directly phosphorylates FOXOs, which leads to FOXO inactivation and retention in the cytosol [19]. Akt activation also results in the down-regulation of GABARAPL1 [15]. Therefore, we addressed whether the activation of Akt could promote LNCaP invasiveness through the inactivation of FOXOs and their target gene GABARAPL1. The expression of constitutively-activated Akt (myr-Akt) promoted the phosphorylation of FOXO1 and FOXO3 and in the meantime decreased the expression of GABARAPL1 (Figure 3G). Moreover, the expression of constitutively activated Akt increased invasion of LNCaP cells (Figure 3H). Forced expression of GABARAPL1 blunted the myr-Akt-enhanced invasiveness of LNCaP cells (Figure 3I). Taken together, these data indicate that the activation of Akt inhibits FOXOs and their target gene GABARAPL1 and promotes CaP invasion.

### Akt expression is negatively related with GABARAPL1 expression in human CaP tumor tissues

**Figure 4:**
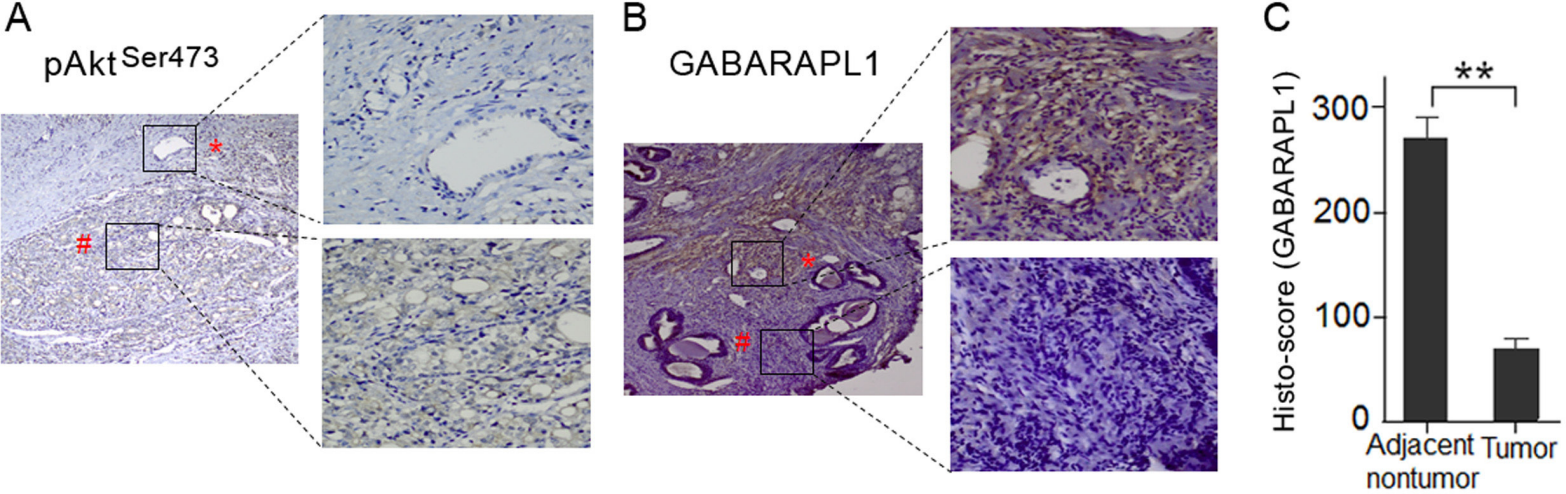
Akt expression is negatively correlated with GABARAPL1 expression in human CaP tumor tissues (**A**) Phosphor-Akt (Ser473) level was assessed in primary human CaP tumor tissues and matched adjacent nontumor tissues by IHC. Representative images of the IHC study were presented. Magnification: image on the left, ×100; images on the right, ×400. (**B**) GABARAPL1 protein expression was examined in primary human CaP tumor tissues and matched adjacent nontumor tissues by IHC. Representative images of the IHC study were presented. Magnification: image on the left, ×100; images on the right, ×400. (**C**) The relative GABARAPL1 histo-scores in tumor tissues and adjacent non-tumor tissues were calculated as described in Methods and presented in a bar graph. **P < 0.01.

To investigate whether the expression of Akt and GABARAPL1 is correlated in clinical samples, we assessed their expression in ten pairs of primary CaP tumor tissues and matched adjacent non-tumor tissues by immunohistochemistry staining. Intense staining of p-Akt^Ser473^ was observed in CaP tumor tissues, while no positive staining was observed in adjacent non-tumor tissues (Figure 4A). In contrast, very low GABARAPL1 staining was observed in CaP tumor tissues compared to high expression in adjacent non-tumor tissues (Figure 4B). The relative GABARAPL1 expression histo-scores in tumor tissues and adjacent non-tumor tissues were calculated by the multiplication of the intensity and area of the IHC staining (Figure 4C). The finding that the expression of Akt and GABARAPL1 is inversely associated in CaP tumor tissues suggests that the inactivation of GABARAPL1 by Akt, most likely through FOXOs, is involved in CaP progression.

## DISCUSSION

There is conclusive evidence that PI3K/Akt pathway plays critical roles in CaP progression and metastasis [6]. As a result, many inhibitors targeting this pathway are now being tested in clinical trials. However, the therapeutic benefit of inhibitors of PI3K, Akt, or mTOR has been modest [8]. The treatment with anti-androgen receptor inhibitor, alone or in combination with PI3K/Akt/mTOR pathway inhibitor such as mTOR inhibitor, has been disappointing in castration-resistant prostate cancer (CRPC). In a recent phase II trial, the combination of carboplatin and everolimus, an mTOR inhibitor, showed minimal efficacy in metastatic CRPC [20]. These results suggest that other mechanisms may also be associated with CaP metastasis.

Vogelstein's group presented a surprising finding that genetic inactivation of both Akt1 and Akt2 resulted in a striking decrease in liver metastasis in a colon cancer mouse model, which correlated with the activation of FOXO proteins, but not the well-known downstream signaling target GSK3b nor mTOR [21]. In line with this finding, our previous *in vitro* screening identified FOXO4 as a metastatic suppressor through the disruption of the PI3K/Akt pathway [10]. The FOXO family members, FOXO1, FOXO3 and FOXO4, are ubiquitously expressed transcription factors that function as tumor suppressors through inhibiting the expression of genes promoting proliferation, survival or de-differentiation [22, 23]. Increasing evidence supports anti-tumor roles for FOXO members in CaP. For instance, the loss of FOXO3a promotes cancer formation in the TRAMP CaP mouse model [24], whereas the upregulation or activation of FOXO proteins leads to growth arrest and apoptosis in CaP cells [25–27]. In contrast, Akt directly phosphorylates FOXO family members, thereby antagonizing the functions of FOXOs by promoting their association with 14-3-3 proteins and thus preventing their nuclear translocation [28]. The cytosol retention of FOXOs leads to their ubiquitylation-mediated proteasome degradation [29]. These observations indicate that Akt/FOXOs pathway may be associated with CaP progression and metastasis.

Our current study identified GABARAPL1 as a potentially metastasis suppressor using a genome wide shRNA screen for increased LNCaP lymph node metastasis *in vivo*. Growing findings indicate that GABARAPL1 is a negative regulator of cancer progression. Decreased GABARAPL1 expression has been reported in various cancer tissues compared to normal tissues [30]. GABARAPL1 expression level is associated with higher histological grade and lower risk of metastasis in breast adenocarcinoma patients, specifically for lymph node-positive patients [31]. These data suggest that GABARAPL1 may play a novel autophagy-independent role as a tumor metastasis suppressor, although it was first identified as an autophagy-related gene.

Our results further show that silencing FOXOs leads to decreased GABARAPL1 expression and increased invasion in LNCaP cells. Constitutively-activated Akt (myr-Akt) increased the invasion of LNCaP cells associated with inactivation of FOXOs and decreased GABARAPL1. Indeed, forced expression of GABARAPL1 partially reversed the increased invasiveness of LNCaP/myr-Akt cells.

Recently, a growing body of work [32–35] has revealed that autophagy promotes multiple steps in the metastatic cascade, such as regulation of the epithelial–mesenchymal transition (EMT), tumor cell migration and invasion. Future studies are needed to elucidate exactly how GABARAPL1 inhibits prostate cancer cell invasion through autophagy pathway. It is unsurprising that the GABARAPL1 inhibited-invasion has been linked to autophagy.

**Figure 5:**
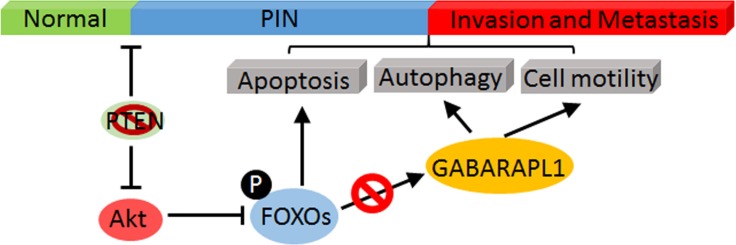
A schematic model of Akt-mediated negative regulation of FOXOs/GABARAPL1 in CaP progress and metastasis Activated Akt directly phosphorylates FOXOs and leads to their inactivation, which subsequently inhibits the expression of their target gene GABARAPL1 and results in enhanced CaP invasion and metastasis.

In summary, our *in vitro* and *in vivo* RNAi screening results strongly suggest that CaP metastasis is promoted by Akt-mediated inhibition of FOXOs activation and the expression of its downstream targets such as GABARAPL1. Hence, we hypothesize that the inhibition of FOXOs-GABARAPL1 signaling by Akt is an important mechanism for CaP progression and metastasis (Figure 5).

## MATERIALS AND METHODS

### Antibodies and reagents

The following primary antibodies (Ab) were used in this study: rabbit polyclonal Abs specific for pan-Akt (#ab8805), p-Akt (Ser473) (#ab81283), anti-GABARAPL1 (#ab86503), and SP rabbit HRP kit were purchased from Abcam (Cambridge, MA). Anti-FOXO1 (#2880), FOXO3 (#12829), FOXO4 (#9472), p-FOXO1 (Ser256) (#9461), and p-FOXO3a (Ser253) (#9466) were from Cell Signaling Technology (Beverly, MA). Mouse monoclonal anti-GAPDH and β-actin was from Santa Cruz Biotechnology (Santa Cruz, CA).

### Cell culture

Human CaP LNCaP cells infected with the DECODE (OpenBiosystems) pooled pGIPZ lentivirus library encoding human shRNAs were established as previously described [10]. LNCaP, PC-3, CWR22Rv1, RWPE-1, and DU145 were purchased from the Chinese Academy of Cell Bank. RWPE-1, LNCaP, LNCaP/LN3, and CWR22Rv1 cells were cultured in RPMI 1640 media supplemented with 10% FBS and incubated at 37°C in a humidified incubator containing 5% CO2. PC-3, and PC-3M/LN4 cells were cultured in DMEM/F12 media supplemented with 10% FBS. DU145 cells were cultured in DMEM media supplemented with 10% FBS.

### Clinical samples

A total of 80 CaP tissue samples and 59 control prostate tissue samples from BPH patients were obtained by needle biopsy in the Department of Urology in Huashan Hospital and the Department of Urology in Cancer Hospital affiliated with Fudan University (Shanghai, China) between March and September 2013. Ten pairs of primary tumor and matched adjacent non-tumor tissue samples were obtained from the Affiliated Hospital of Xuzhou Medical College (Xuzhou, Jiangsu, China). Written consents were obtained from all subjects prior to the recruitment. The study protocol was approved by the Ethics Committee of Huashan Hospital, Cancer Hospital of Fudan University, and the Affiliated Hospital of Xuzhou Medical College. All tumor samples were confirmed to contain more than 80% tumor cells by histological examination of sequential sections by pathologists. Staging was assessed after pathological examination of formalin fixed specimens according to the 1997 TNM classification. The clinical and pathologic characteristics of the subjects were reported previously [36].

### Transient transfection

pLKO.1-shRNA-GABARAPL1 plasmid [37], constitutively-activated Akt1 (myr-Akt1) or control vectors were transiently co-transfected with pEGFP DNA (Clontech/Takara, Mountainview, CA) into LNCaP cells using Lipofectamine reagent (Invitrogen, Carlsbad, CA) according to the manufacturer's protocol. pAd-GABARAPL1 plasmid [37] was co-transfected transiently with pEGFP DNA into CWR22Rv1 cells. The transfected LNCaP and CWR22Rv1 cells were incubated for 40 h. GFP positive cells were sorted by a fluorescence-activated cell sorter and used for Western blotting and invasion assays.

### Orthotopic CaP metastatic model

Testosterone pellets were embedded in the flanks of 6–8 weeks old male nude mice (five mice per group) as previously described [10]. 10^6^ of LNCaP cells infected with GFP-labeled lentivirus library encoding human genomic shRNAs (13,650 targeted genes in 7 pools of 9,750 shRNA clones/pool) were orthotopically injected into the dorsal lobes of the prostate of these male nude mice as previously described [10]. Ten weeks later, mice were sacrificed and examined for the presence of GFP fluorescence in primary tumors and the organs using whole-body imaging (Lightools Research, Encinitas, CA). Pelvic lymph nodes were collected and digested with proteases. The cell suspension was placed on culture dishes and selected for puromycin (2 μg/ml) resistant metastatic LNCaP cells. All animal protocols were approved by the Institutional Animal Care and Use Committee of Shenzhen Biochemical Institute.

### siRNA transfection

Synthetic ON-TARGETplus SMARTpool siRNA specific for FOXO1, FOXO3 or FOXO4, siCONTROL non-specific siRNA (NS-siRNA), and DharmaFECT-1 transfection reagent were purchased from Dharmacon (Lafayette, CO). LNCaP cells were plated in 6-well plates (5 × 10^4^ per well) overnight. Cells were transfected with 50 nM of NS-siRNA or three FOXO-specific siRNAs for 24 h using DharmaFECT1 following the manufacturer's protocol.

### Immunoblot (IB) analysis

The cells were lysed in RIPA buffer with protease inhibitor cocktail (Roche Diagnostics, Mannheim, Germany). Protein samples (40 μg) were separated by SDS-PAGE, blotted onto polyvinylidene fluoride membranes which were blocked for 30 min with 5% bovine serum albumin (Sigma) in 1 × TBS/T (0.1% Tween-20 in Tris-buffered saline) and then probed with primary antibodies as indicated. Digital imaging and signal quantification were performed on a Chemi-Genius2 Bio-Imager (Syngene, Frederick, MD) using GeneTools software.

### Invasion assay

Modified Matrigel-based Boyden chamber assays were performed as previously described [38]. Briefly, LNCaP or CWR22Rv1 cells transfected with plasmids or siRNAs were plated in the inserts of the invasion chambers. FBS (10%) was added as a chemoattractant. After 24 h of incubation, cells that had not penetrated the membranes were removed from the inserts with cotton swabs. Chambers were fixed and stained using Diff-Quik Stain Set (Dade Behring Inc., Newark, DE) and examined under a bright-field microscope. Numbers of invaded cells were counted in 6 fields per membrane (×20 objective) and the average values of three independent experiments were presented.

### Immunohistochemistry (IHC) and imaging

IHC assays were performed as previous described [36]. Briefly, ten pairs of primary tumor and matched adjacent non-tumor tissue samples were obtained from patients after prostatectomy and fixed in 10% buffered formalin, embedded in paraffin and sectioned at 4 microns. Slides were deparafinized in several baths of xylene and then rehydrated in graded alcohol series followed by ddH2O. Primary Abs to p-Akt(Ser473) (1:500) and GABARAPL1 (1:500) were diluted in 1% BSA solution and incubated for 1 h at 37°C, followed by staining using HRP-conjugated secondary antibody for 1 h at room temperature. Slides were scanned with Olympus BX53 microscope and viewed using Cellsens Entry (Olympus). The intensity of immunoreactivity (intensity score) was judged on an arbitrary scale of 0–4: 0, negative; 1, weak; 2, moderate; 3, strong and 4, very strong. Additionally, samples were given a percentage score based on the percentage of tissue area displaying each intensity score. A histo-score was calculated for each sample by multiplying the intensity score and the percentage score. Thus, the histo-score ranges from 0 to 400.

### Quantitative reverse transcriptase PCR (qRT-PCR)

Total RNA was isolated using TRIZOL Reagent (Sigma) and was reverse transcripted into cDNA using Revert Aid First Strand cDNA Synthesis Kit (Thermo Scientific). Real-time PCR was performed using SYBR premix EX Taq (TaKaRa) and analyzed with CFX96 Real-Time System (BIO-RAD). PCR primer sequences are as follows: GABARAPL1: forward, 5′-AGGAGGACCATCCCTTTGAGT-3′; and reverse, 5′-TGGCCAACAGTAAGGTCAGA-3′. β-actin: forward, 5′-GACCTGACTGACTACCTCATGAAGAT-3′; and reverse, 5′-GTCACACTTCATGATGGAGTTGAAG G-3′. β-actin was used as a housekeeping gene for the qRT-PCR reactions. Each test was done in triple replication and the 2^−ΔΔCt^ method [26] was used to calculate the expression of genes.

### Statistical analyses

Statistical significances between groups were determined by two-tailed student's *t*-test. The differences in gene expression levels between CaP tumor tissues and BPH tissue specimens were tested by chi-square test. All statistical analyses were performed by using SPSS 16.0 software program. *P* < 0.05 was considered statistically significant.

## Abbreviations

CaP: prostate cancer
CRPC: castration-recurrent prostate cancer
GABARAPL1: gamma-aminobutyric acid (GABA) A receptor-associated protein-like 1
ATG8: autophagy-related 8
GEC1: Glandular epithelial cell protein 1
FOXOs: FOXO transcription factors
BPH: benign prostate hyperplasia
LEPRE1: Leucine proline-enriched proteoglycan(leprecan)1
MYO3B: Myosin IIIB
ZNF763: zinc finger protein 763
TRDMT1: tRNA aspartic acid methyltransferase 1
OGFRL1: Opoid growth factor receptor-like 1
DYX1C1: Dyslexia susceptibility 1 candidate 1
qRT-PCR: real-time quantitative RT-PCR
LN: lymph nodes
PI3K: Phosphatidylinositol-3-kinase
IHC: Immunohistochemistry
myr-Akt1: Akt1 activation
EMT: epithelial–mesenchymal transition
